# Spatial heterogeneity of heavy metal contamination in soils and plants in Hefei, China

**DOI:** 10.1038/s41598-018-36582-y

**Published:** 2019-01-31

**Authors:** Qianjin Zhang, Ruoyun Yu, Songling Fu, Zemin Wu, Han Y. H. Chen, Hua Liu

**Affiliations:** 1 School of Forestry & Landscape Architecture, Anhui Agricultural University, Hefei, 230036 China; 2 Faculty of Natural Management, Lakehead University, Thunder Bay, ON P7B5E1 Canada

## Abstract

The contamination of soil and plants with heavy metals, which has detrimental influences on plant growth, water purification, and food safety, has emerged as a serious global issue. To better understand the spatial variations of contamination of heavy metals associated city development and land use types, we collected soil samples and *Magnolia grandiflora* branches to quantify lead (Pb) and cadmium (Cd) contents of the roadside, industrial, residential, and park greenbelts in Hefei City, China. We found that Pb content in soil was the highest in roadside greenbelts and the lowest in parks with industrial and residential greenbelts being intermediate, while Cd in soil was the highest in greenbelts close to city center and decreased with the distance to city center. Pb in *M. grandiflora*, however, did not differ among greenbelt types but decreased with distance to the city center. Cd in *M. grandiflora* was the highest in roadside and lowest in parks and also decreased with the distance to the city center. Across all greenbelt types and the distances to the city center, Pb and Cd contents were positively correlated in soil and plants. Our findings suggest that vehicle traffic, population density, and age of urbanization collectively contribute to soil and plant contamination of Pb and Cd.

## Introduction

As urbanization accelerates, anthropogenic activities introduce large volumes of contaminants into the urban environment, provoking severe heavy-metal pollution in urban soils^1,2^. This has been recognized as a major concern at local, regional and global levels due to the impacts of the contaminants on human health^3^ as well as their disruption to the geochemical cycling of the urban ecosystems^4^. Therefore, it is imperative to evaluate and quantify the heavy metal contamination of urban soils and plants as well as to understand their spatial distributions in urban environments.

Recent advances have been made in the understanding of the soil contamination of heavy metals^5–7^ as well as annual variations in both industrial and urban soils^3^. Zhao and Hazelton^7^ showed apparent correlations between the contents of Pb and Zn with the distance from the roadside and depth in three soil types in Miranda Park, Sydney. The highest Pb contamination often occurs in the top (0–20 cm) soil layer near roadsides in Warsaw, Poland^6^. In New Zealand, soils in native urban forests have the lowest pools of heavy metals compared with green spaces close to high-traffic, park, school, industrial and residential areas^8^. China has experienced the highest rate of urbanization in the world during the past four decades^9^. Although recent studies have shown trace and heavy metal contamination in urban soils in China but see^5,10^, our understanding of the impact of China’s urbanization on heavy metal contamination in urban soils remains incomplete.

Pb and Cd have high chemical activity in soil, which translates to the easy absorption by plants and ultimately threatens human health through the food chain^7,11,12^. For example, the contents of heavy metals in mulberry fruit are strongly correlated with the effective contents of Pb, Cr, Co, Zn, Mn, Cu, and Ni in the soil^13^. Excessive amounts of heavy metals in soils can disturb plant growth and development and alter plant community structure. For example, soil contamination of Zn, Cd, and Cu has a strong degradative effect on plant diversity and reduce population sizes in grasslands^14^. Cd and Pb are not essential nutrients for plants, but they can damage organisms at low contents via their impact on cell growth and division, plant photosynthesis, the uptake of water, and plant respiration^15–18^.

Urban greenbelts play a critical role in air purification and the phytoremediation of heavy metal pollution^19^. Plants employ dual strategies for the absorption of heavy metals; one is their interception and absorption from the atmosphere through their leaves^20^, while the other is through their root systems^21,22^. For example, an adaptation mechanism of plant species such as *Equisetum ramosisti* and *Leersia hexandra* may balance the absorption and transfer of Pb, Zn, and Cu under extreme pollution conditions^23^. In the investigation of plants and heavy metals, a large number of annual herbs and other crops were planted and harvested for the control of soil pollution^24–27^. However, compared with herbaceous plants, woody plants have deeper roots, larger biomass and longer growth cycle characteristics, which may thus have more long-term positive impacts on the remediation of heavy metal contamination. A recent study has shown that the responses of urban woody plants to heavy metals have important roles in the development of urban greening and the remediation of heavy metals in urban soils^28^.

Here we attempted to understand the extent of Cd and Pb contaminations in Hefei and its spatial variation. This is mainly because the two metals of Cd and Pb are major pollutants in the city. Like most large cities in China^9^, Hefei has experienced a rapid expansion in the past four decades. As an important part of city greening, four types of green space account for the majority of green spaces in the city, including roadside, industrial, and residential greenbelts and parks. In these green spaces, *Magnolia grandiflora* L., a tree species extensively distributed across Southern China, was designated as the city tree in 1984 and has been a major greening tree species in city green spaces. We hypothesized that (1) heavy metal contamination would differ among greenbelts with the lowest in parks and the highest along roadsides in both soil and *M. grandiflora* plants since vehicle wastes are major sources of heavy metals in urban environments^29^; (2) heavy metal contamination would increase with distance from the center of city because the city has expanded from the city center and more advanced establishments have higher contamination^10^; (3) that the Cd and Pb contents in *M. grandiflora* plants would increase with those in the soil. We expect that our results help better understand the impacts of urbanization and land use on the spatial patterns of heavy metal contamination, and contribute to the identification of the potential application of trees for soil phytoremediation.

## Results

**Figure 1:**
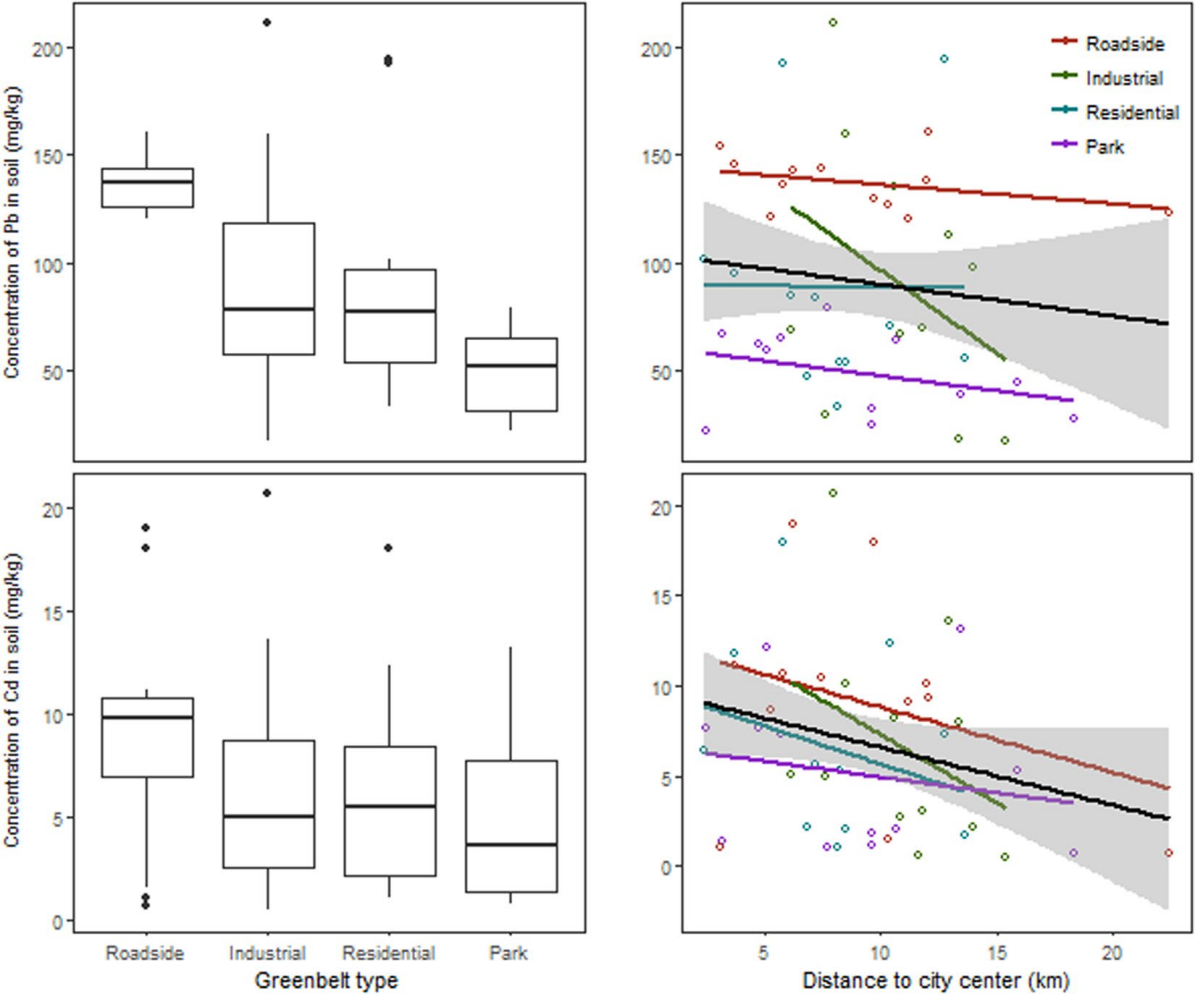
The contents of Pb and Cd (mg/kg) in soils in relation to greenbelt type and distance to city center in Hefei city. (**A**) boxplots showing the effects of greenbelt types, (**B**) The effects of the distance to city center. Values for boxplots are medians, 75% observations in boxes, and whiskers above and below the box indicate 95^th^ and 5^th^ percentiles. In (**B**), open points are individual observations. Lines and shades are fitted regressions with 95% confidence intervals.

Soil resident Pb content varied among greenbelt types (*p* < 0.001) with a similarly marginal decline with the distance from city center (*p* = 0.165) (Table 1, Fig. 1A,B). Among greenbelt types, roadside greenbelts had more than two-fold Pb than those in parks, while industrial and residential greenbelts were intermediate (Fig. 1A). Soil Cd content decreased significantly with the distance from city center (*p* = 0.017) in a similar manner among greenbelt types (*p* = 0.720), but the trend of decreases from the roadside to park greenbelts was not statistically significant (*p* = 0.486) (Table 1, Fig. 1A,B).

**Table 1:** The effects of greenbelt types (T) and distance to city center (D) on Cadmium (Cd) and lead (Pb) contents (mg/kg) in soils and plants.

Model	Source	Df	Sum Sq	F	P
Pb in soil	T	3	7.54	8.82	<0.001
	D	1	0.57	2	0.165
	T × D	3	0.78	0.91	0.443
	Residuals	40	11.4		
Cd in soil	T	3	2.59	0.83	0.486
	D	1	6.45	6.19	0.017
	T × D	3	1.4	0.45	0.720
	Residuals	40	41.67		
Pb in *M. grandflora*	T	3	0.15	0.45	0.719
	D	1	0.57	4.99	0.031
	T × D	3	0.05	0.15	0.931
	Residuals	40	4.53		
Cd in *M. grandflora*	T	3	7.91	5.53	0.004
	D	1	1.87	3.92	0.057
	T × D	3	0.65	0.46	0.714
	Residuals	32	15.26

**Figure 2:**
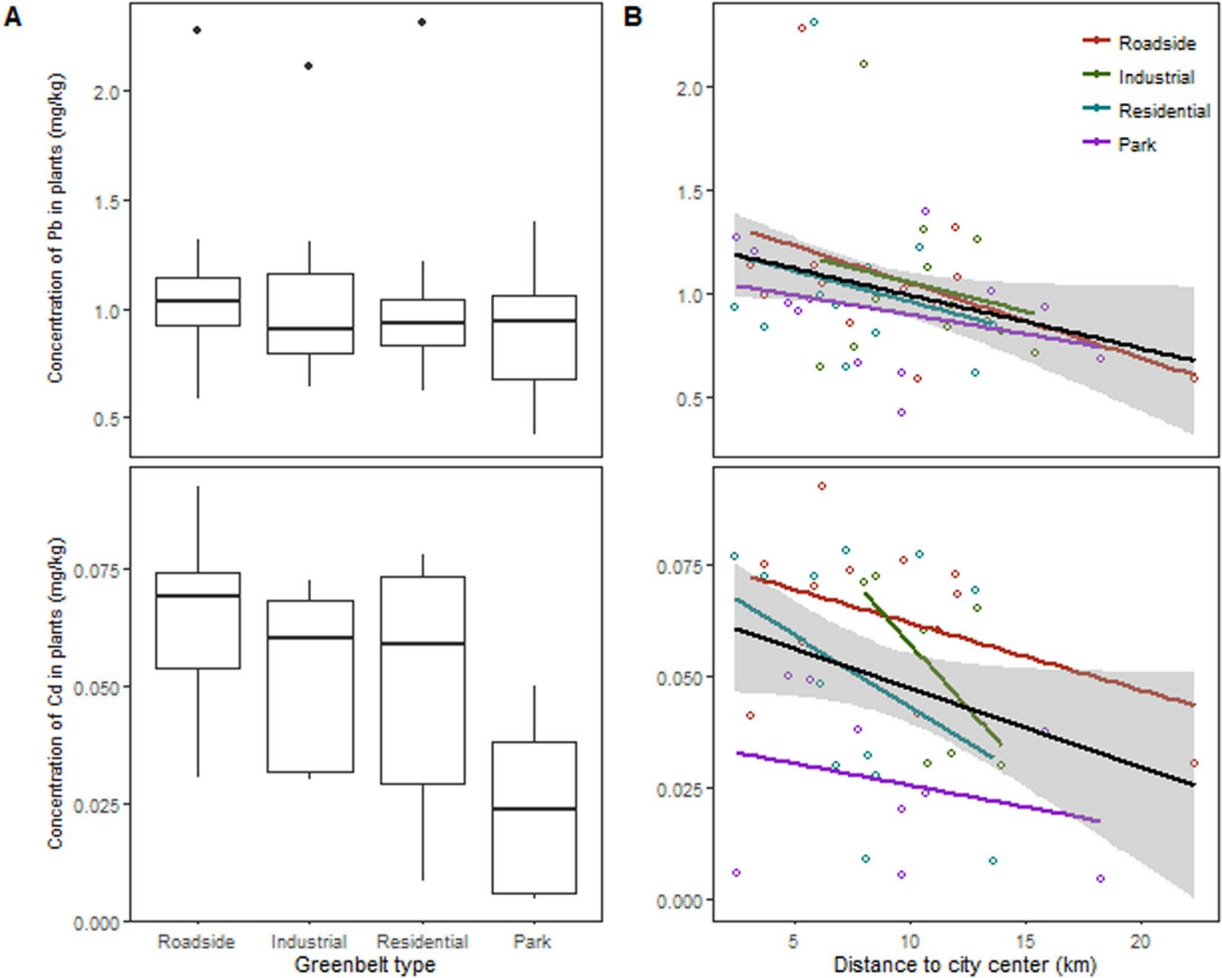
The contents of Pb and Cd of *M. grandflora* in relation to greenbelt type and distance to city center in Hefei city. (**A**) boxplots showing the effects of greenbelt types, (**B**) The effects of the distance to city center. Values for boxplots are medians, 75% observations in boxes, and whiskers above and below the box indicate 95^th^ and 5^th^ percentiles. In (**B**), open points are individual observations. Lines and shades are fitted regressions with 95% confidence intervals.

The Pb content of *M. grandflora* decreased significantly with the distance from city center (*p* = 0.031) in all greenbelt types (*p* = 0.931) with no significant difference among greenbelt types (*p* = 0.719) (Table 1, Fig. 2A,B). The Cd content of *M. grandflora *differed among greenbelt types (*p* = 0.004) and decreased marginally with the distance from city center (*p* = 0.057) (Table 1, Fig. 2A,B). Among greenbelt types, parks had significantly lower Cd content than other types (Fig. 2A).

**Figure 3:**
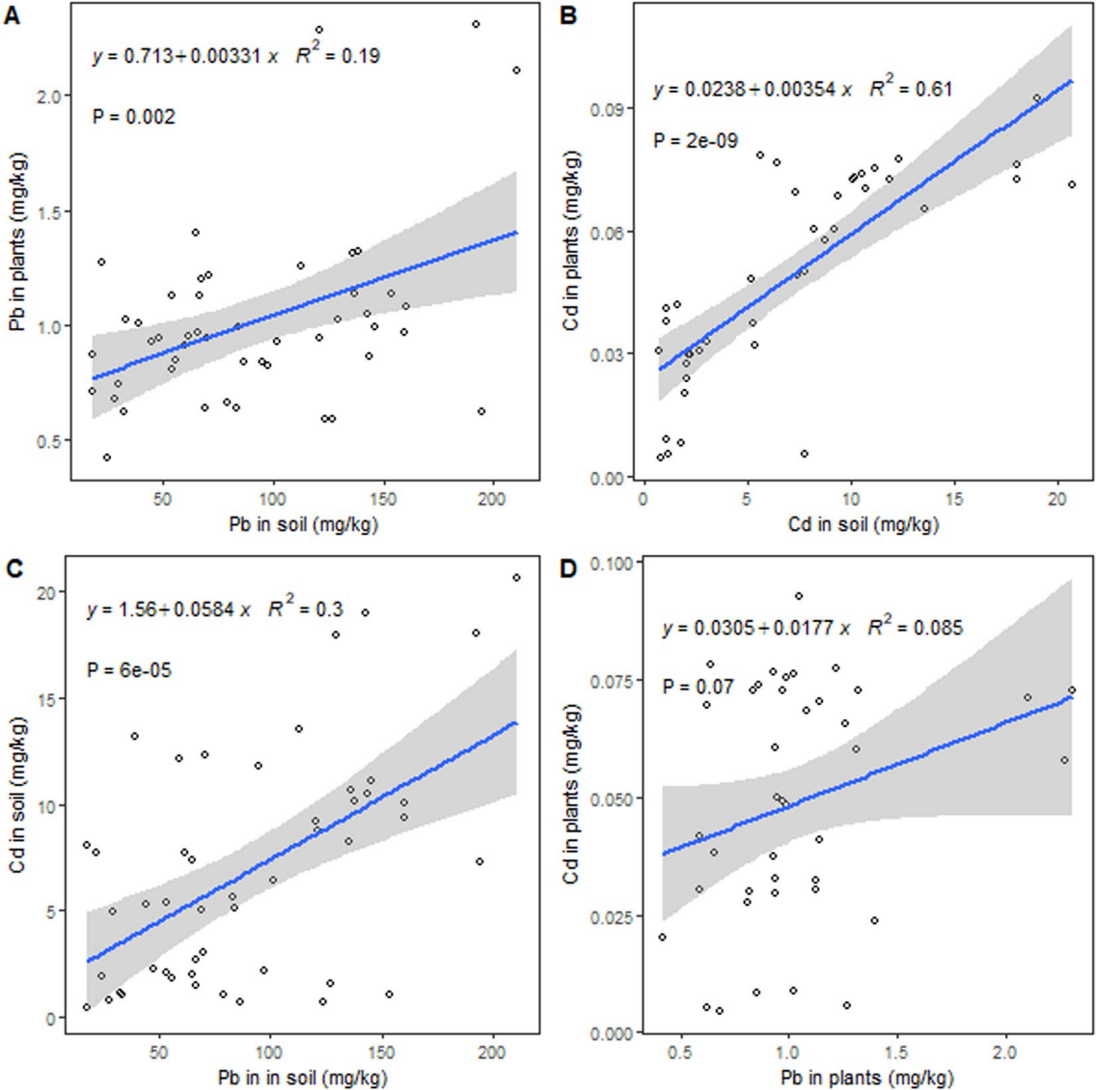
Relationship between Pb and Cd contents in soil and plants. (**A**) The Pb contents in soil and plants, (**B**) The Cd contents in soil and plants, (**C**) The Cd content in relation to the Pb content in soil, and (**D**) The Cd content in relation to the Pb content in plants.

Regression analysis showed that both Pb and Cd contents in *M. grandiflora* were strongly related to their respective contents in soil (*p* = 0.002 and <0.0001, respectively) (Fig. 3A,B). Moreover, the Pb content in soil was significantly correlated to the Cd content in soil (Fig. 3C), while there was a marginally positive correlation between the contents of Pb and Cd in plants (Fig. 3D).

## Discussion

Our results revealed that Pb and Cd contaminations were spatially variable. In soil, Pb contamination was more strongly associated with greenbelt types than the distance to the city center, but Cd contamination was more predictable by the distance to the city center. In *M. grandflora*, Pb contamination decreased with the distance to the city center, while Cd contamination decreased with the distance to city center and differed with greenbelt types. Across all sites, Pb and Cd contaminations in soil and plant were positively correlated.

Our results revealed that the Pb content in the soil of roadside greenbelts was higher than that of industrial, residential, and park greenbelts, suggesting that vehicle traffic is a major source for the contamination of Pb in the soil. Our finding is consistent with the previous results indicating that atmospheric transport is a significant determinant for heavy metal in soils across a range of land use types^30–33^. Similarly, McCumber and Strevett^34^ reported that heavy metal deposition by air traffic for 50–90% of the heavy metal increases in soils, with a strong positive correlation between soil resident Pd contents and distance from an airport. Our result of an insignificant difference of Cd among greenbelt types, which is similar to a previous finding^35^, may suggest that Cd is more mobile than Pd^36^. Alternatively, observed Cd contents in soil reflect its long-term accumulation of rather than the current vehicle traffic.

We found that Cd content, and to a lesser degree, Pd contents in soil decreased with the distance to city center across a range of greenbelt types. While it is well recognized that heavy metals contaminations are spatially variable in urban districts^3,7,10,12,32,37,38^, our finding indicates that the extent of soil heavy metal contamination increases with urbanization age. The higher population density, coupled with more traffic and more wastes, shall also a contributor to higher soil contamination in city centers. Moreover, because both heavy metals have long residual times, their higher concentrations in the city center than the more recently developed urban landscapes are attributable to their longer accumulation in soils and higher population density.

We found the Pb and Cd contents in *M. grandiflora* branches decreased with the distance to city center and that the Cd content was the highest in the branches of *M. grandiflora* trees at roadside greenbelts and the lowest in parks. The somewhat different responses between the Pb and Cd contents in soil and plants to greenbelt types may reflect that the contents of heavy metals in plants are also influenced by their particular physiological characteristics^39,40^. Nevertheless, our regression analysis indicated that there were strong positive correlations between the heavy metal contents in soils and those of *M. grandiflora* branches. This indicates that *M. grandiflora* plants uptake more heavy metals with increasing soil contamination, indicating that the heavy metal contents in soil strongly influence the heavy metal content of plants.

Concurrently, our results indicate that *M. grandflora* possess strong phytoextraction capacities. Woody plants accumulate high amounts of heavy metals, and the capacities are different among species^41^. These were confirmed by five tree species grown in Yantai city, China^42^. In particular, *M. grandflora* branches have a higher accumulation capacity for most heavy metals than leaves^43^. We found that the contents of Pb and Cd in *M. grandflora* branches were on average 1.014 mg/kg and 0.049 mg/kg, and approximately up to 2 mg/kg and 0.8 mg/kg, respectively, which are higher than those in many other species^41–43^. Together, these results indicate that *M. grandiflora* woody tissues possess a high capacity for heavy metals absorption, such that it may grows normally in a polluted environment, and is thus a pollution-tolerant plant species. The specific physiological and ecological characteristics of *M. grandiflora* will require further investigation. Nevertheless, because it was not sensitive to heavy metals, and is a beautifying contributor to city greening, *M. grandiflora* might be effectively employed as an appropriate pioneer species toward the vegetative remediation of polluted urban areas.

## Conclusion

By investigating the spatial variations of Pb and Cd contents in soils and *M. grandiflora* branches in Hefei city, we show that Pb contents in soil was positively associated with vehicle traffic and increased with urbanization age as well as population density, while Cd contents in soil were more strongly influenced by urbanization age and population density. Across the greenbelt types and distances to the city center, the contents of Pb and Cd in *M. grandiflora* were positively associated with their contents in soil. This suggests that *M. Grandiflora* has a high capacity to uptake Pb and Cd in contaminated urban soils.

## Materials and Methods

### Study area

**Figure 4:**
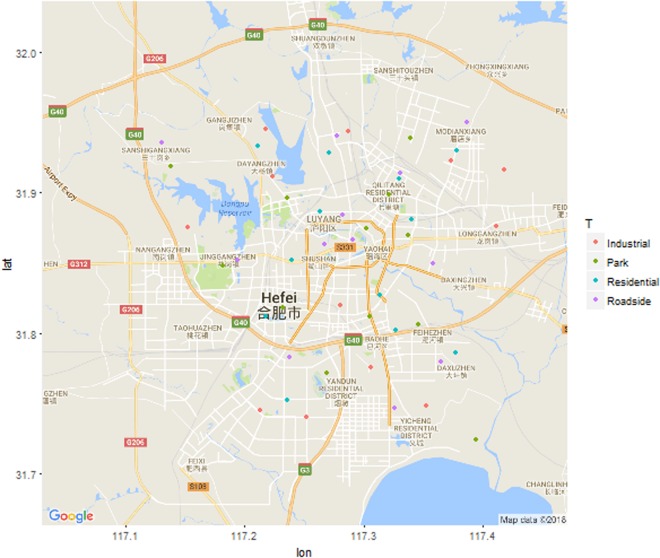
Distribution of sampling plots in Hefei City, China. The map was produced by using the package ‘*ggmap*’ in R (https://cran.r-project.org/web/packages/ggmap/)^47^.

Hefei city (Fig. 4) is located in Anhui Province, in Eastern China, within the lower reaches of the Yangtze River (31°48′~31°58′N, 117°11′~117°22′E). This area belongs to a subtropical humid monsoon climate zone, with an annual average air temperature of 15.7 °C, an annual average rainfall of 1000 mm, with an annual 2100 h of sunshine. In recent decades, Hefei has undergone rapid and dramatic urban renewal and development. From 1949 to 2015, its population increased from 0.053 to 7.69 million, while the built-up area increased from 5.2 km^2^ to 339 km^2^. The zonal vegetation and soil are deciduous and evergreen broad-leaved forests and Alfisol, respectively. The vegetation coverage rate is 39.5%, which allows for a per capita green area of up to 9.3 m^2^. The most abundant trees species for urban greening are *M. grandiflora*, *Cinnamomum camphora*, *Platanus acerifolia*, *Salix matsudana*, *Koelreteria bipinnata*, *Sophora japonica*, *Cedrus deodara*, *Elaeocarpus sylvestris*, *Ginkgo biloba*, *Paulownia fortune*, and *Osmanthus fragrans*. Among them, *M. grandiflora* accounts for 8.7% of the total number of urban trees. Industrial establishments, transportation networks, residential communities, and other support systems are integrated across the urban landscape.

### Sampling

We collected soil samples and branch samples of *M. grandiflora* from the greenbelts in roadsides, industrial areas, residential areas, and parks of four districts (Luyang, Shushan, Yaohai, and Baohe) classified by Hefei city government, and all samples were collected in autumn. To examine the spatial variation, we measured the distance to the city center for each sample site. We established 48 sites across the entire city covering four greenbelt types with a wider range of distances from the city center (Fig. 4). In each sample site, a circular sample plot of 100 m^2^ was randomly established to represent the sampling site. In each sample plot, we randomly allocated three soil pits and dug to a depth of 40 cm, and we took soil samples in equal volumes along the vertical profiles. Three soil samples within each plot were mixed into a composite sample for laboratory analysis. Three health trees of *M. grandiflora* were selected as sample trees from each plot, and then collected branch samples from the canopies of each sample tree in north, south, east, and west orientations, all sample branches were 2-year old and located in outside and middle part of the canopy. The sample trees were visually healthy without visual signs of diseases. The xylems of the sample branches from each sample plot were combined into a composite sample for laboratory analysis. On average, sample trees had a mean tree height of 7.0 ± 0.5 m (s.e.m.) and mean diameter at breast height of 17.8 cm ± 1.0 cm (s.e.m.), which did not differ significantly with greenbelt types (P > 0.05).

### Samples measurement

The soil samples were air-dried at room temperature (23 °C) and then sieved through a 1 mm and 0.15 mm mesh in sequence. A subset of 0.25 g was then digested with HCl –HNO_3_ -HClO_4_ in 50 mL Teflon beaker. The plant samples were rinsed with deionized water to remove the sediment particles and then dried in an oven at 70 °C to a constant weight. The dried plant tissues were subsequently weighed and ground into powder. The plant samples (0.50 g) was carried out through acid digestion, to which a mixture of concentrated HNO_3_ − HClO_4_ (4:1, v/v) was added. All the digested samples of soil and plant were diluted with 25 mL of distilled water. The heavy metals contents of them were measured using Inductively- Coupled Plasma-Atomic Emission Spectrometry (ICP-AES) (ICP 7000DV, USA).

### Statistical analysis

To simultaneously test the effects of greenbelt type and the distance to the city center on Pb and Cd contents in soil and plants, we used the following linear model:

1 $${Y}_{ijk}={T}_{i}+{D}_{j}+T\times {D}_{ij}+{\varepsilon }_{k(ij)}$$

Where *Y*_*ijk*_ is Pb or Cd content in soil or plants, *T*_*i*_ (*i* = roadside, industrial, residential, and park) is greenbelt type, *D*_*j*_ is the distance to the city center in km as a continuous variable, and ɛ_*k*(ij)_ is sampling error. We assessed the model’s assumptions of normality and homogeneity by Shapiro-Wilk’ test and Breusch-Pagan test, respectively. Natural log transformation on Pd and Cd contents helped meeting the normality assumption, but homogeneity was still violated for one of the four tests. To mitigate small sample sizes and violation of the assumptions, we bootstrapped regression parameter estimates by 1000 iterations via package ‘*boot*’^44^ within ‘*ggplot2*’^45^. We compared the bootstrapped estimates with those of the linear models and found that both methods yielded qualitatively similar trends. For simplicity, we reported the linear model results. To examine the association between the Pb and Cd contents between soil and plants, we used linear regression analysis. All analysis was performed using R Statistical Software, version 3.4.1^46^.
